# Shifting Patterns of Influenza Circulation during the COVID-19 Pandemic, Senegal

**DOI:** 10.3201/eid2909.230307

**Published:** 2023-09

**Authors:** Alexandre Lampros, Cheikh Talla, Maryam Diarra, Billo Tall, Samba Sagne, Mamadou Korka Diallo, Boly Diop, Ibrahim Oumar, Ndongo Dia, Amadou Alpha Sall, Mamadou Aliou Barry, Cheikh Loucoubar

**Affiliations:** Hôpital Européen Georges Pompidou, Paris, France (A. Lampros);; Institut Pasteur de Dakar, Dakar, Senegal (A. Lampros, C. Talla, M. Diarra, B. Tall, S. Sagne, M. Korka Diallo, N. Dia, A.A. Sall, M.A. Barry, C. Loucoubar);; Government of Senegal Ministry of Health and Social Action, Dakar (A. Lampros, B. Diop);; World Health Organization, Dakar (A. Lampros, I. Oumar)

**Keywords:** Influenza, COVID-19, SARS-CoV-2, viruses, respiratory infections, severe acute respiratory syndrome coronavirus 2, SARS, coronavirus disease, seasonality, coinfection, viral interference, surveillance, epidemiology, Senegal, Africa

## Abstract

Historically low levels of seasonal influenza circulation were reported during the first years of the COVID-19 pandemic and were mainly attributed to implementation of nonpharmaceutical interventions. In tropical regions, influenza’s seasonality differs largely, and data on this topic are scarce. We analyzed data from Senegal’s sentinel syndromic surveillance network before and after the start of the COVID-19 pandemic to assess changes in influenza circulation. We found that influenza shows year-round circulation in Senegal and has 2 distinct epidemic peaks: during January–March and during the rainy season in August–October. During 2021–2022, the expected January–March influenza peak completely disappeared, corresponding to periods of active SARS-CoV-2 circulation. We noted an unexpected influenza epidemic peak during May–July 2022. The observed reciprocal circulation of SARS-CoV-2 and influenza suggests that factors such as viral interference might be at play and should be further investigated in tropical settings.

In temperate regions, seasonal influenza commonly follows a regular circulation pattern and has an annual epidemic peak during the colder winter months (*1*–*3*). In contrast, tropical areas have great diversity in influenza seasonality (*1*–*3*). Some countries, including Brazil, Mexico, and the Philippines, report 1 distinct annual peak, but other countries, including Colombia, Burkina Faso, and Thailand, have 2 distinct peaks (*3*). Countries near the equator, such as Venezuela, Cameroon, Indonesia, and Malaysia, show year-round circulation and have no distinct peak (*3*). However, Senegal and other countries in West Africa have year-round influenza activity with 1 or 2 distinct annual peaks; the second most often occurs during the rainy season (*3*).

The diversity of circulation patterns challenges old theories on influenza’s seasonality that suggest the increased activity seen in winter mainly is explained by the permissive dry and cold weather (*4*). The determinants of influenza’s seasonality remain poorly understood, and studying viral circulation in tropical areas represents a crucial step toward a global understanding of influenza seasonality (*2*,*5*–*7*).

The emergence of SARS-CoV-2 in late 2019 deeply impacted influenza’s global circulation (*8*). During 2020–2021, the first years of the pandemic, historically low levels of influenza circulation were noted, but those findings were largely described and discussed from high-income countries in temperate regions that have abundant influenza surveillance data (*9*–*12*). The low-level phenomenon is commonly believed to be a beneficial side effect of nonpharmaceutical interventions (NPIs) implemented to control of the spread of SARS-CoV-2 (*13*,*14*). However, little is known about the impact that SARS-CoV-2 had on influenza’s circulation in tropical settings. To clarify SARS-CoV-2–influenza interactions in tropical regions, we investigated usual influenza circulation patterns in Senegal, a subtropical country in West Africa, and whether circulation patterns shifted during the COVID-19 pandemic.

## Methods

### Syndromic Surveillance in Senegal

Since 2011, Senegal has been managing a sentinel syndromic surveillance system (réseaux de surveillance sentinelle syndromique du Sénégal), known as the 4S Network (*15*). The 4S Network is concurrently run by the National Ministry of Health and Institut Pasteur de Dakar, which supervises the sites’ activities, provides equipment, and manages sample transport, virological testing, and data management and analysis.

**Figure 1:**
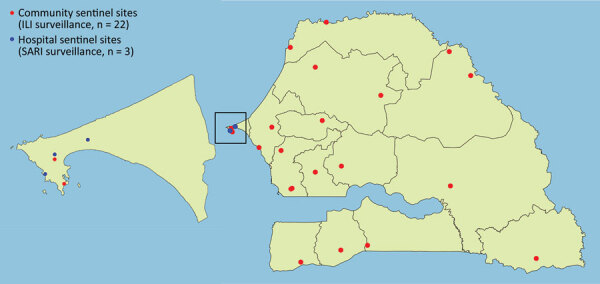
Geographic distribution of community and hospital sentinel sites participating in surveillance for shifting patterns of influenza circulation during the COVID-19 pandemic, Senegal. Sites represent the network of sentinelle syndromique du Sénégal (sentinel syndromic surveillance of Senegal), also known as the 4S Network. Enlarged map at left shows detailed view of the Dakar capital region and 4S Network hospitals located in the region. ILI, influenza-like illness; SARI, severe acute respiratory illness.

The 4S Network functions as any syndromic surveillance system by monitoring and testing persons who have certain syndromes of public health interest, in this case, signs and symptoms suggestive of viral respiratory diseases, as previously described (*16*). The 4S Network comprises 25 sentinel sites: 22 community sites in primary or secondary healthcare facilities that are in charge of influenza-like illness (ILI) surveillance and 3 hospitals located in the region of Dakar that are in charge of severe acute respiratory illness (SARI) surveillance (Figure 1). Sentinel sites are located throughout the country in each of its 14 regions, enabling geographic coverage and providing a fairly accurate representation of Senegal’s population. Sites were selected according to their location, number of patients served, willingness to participate, and availability of minimal equipment, such as running water and a refrigerator (*16*).

The 4S Network offers a unique source of epidemiologic data on ILI and SARI in Senegal. During the COVID-19 pandemic, the network also rapidly integrated SARS-CoV-2 testing in its routine surveillance activities. We extracted data from the 4S Network to analyze local dynamics of influenza, SARS-CoV-2, and interactions between the 2 viruses in a remote setting.

### Study Population and Case Definition

We focused on ILI and SARI surveillance by using definitions from 2014 World Health Organization criteria (*17*). Those criteria define ILI cases as acute respiratory infection accompanied by a measured temperature of ≥38°C and cough that had an onset within the previous 10 days and define SARI cases as an acute respiratory infection and history of fever or a measured temperature of ≥38°C and cough that had an onset within the previous 10 days and resulted in hospital admission. We included all age groups in the study and had no specific exclusion criteria apart from a patient’s refusal to participate. All patients undergoing virological testing and included in the surveillance program gave informed oral consent. All data were fully anonymized in advance.

### Study Period and Data Collection

To assess baseline influenza seasonality patterns, we extracted influenza test results from January 1, 2013–March 1, 2020. To describe interactions between SARS-CoV-2 and influenza, we extracted those test results from March 1, 2020–July 31, 2022.

For SARI cases, any patient that fit the case description and was admitted at a sentinel site was subjected to nasal and oropharyngeal swab sampling. For ILI surveillance, ≥5 samples per site were randomly collected for surveillance every week. SARI samples are transferred every day and ILI samples are transferred weekly to the national reference center for influenza and other respiratory viruses at the Pasteur Institute in Dakar.

The reference center performs 2-step real-time reverse transcription PCR (rRT-PCR) by using the CFX96 Real-Time PCR Detection System (Bio-Rad, https://www.bio-rad.com) and Anyplex II RV16 Detection Kit (Seegene, https://www.seegene.com). That testing system enables simultaneous testing for influenza A and B viruses; human respiratory syncytial virus A and B; adenovirus; metapneumovirus; coronavirus 229E, NL63, and OC43; parainfluenza virus 1−4; rhinovirus A/B/C; enterovirus; and bocavirus (*18*). Influenza viruses underwent RT-PCR to detect N1, H1, and H3 subtypes and matrix, neuraminidase 2, and hemagglutinin 2 genes, as previously described (*19*).

SARS-CoV-2 surveillance was rapidly integrated into the 4S Network. At the beginning of June 2020, every sample from SARI or ILI cases was subjected to monoplex SARS-CoV-2 RT-PCR testing by using the LightMix CoV E-gene and LightMix Modular Wuhan CoV RdRP-gene kits (TIB MOLBIOL, https://www.tib-molbiol.de). Although a new case definition including other symptoms, such as anosmia or digestive symptoms, for suspected COVID-19 cases was initially added to the surveillance system, ILI and SARI case definitions remained unchanged during that period. Senegal abandoned the new suspected COVID-19 case definition at the end of 2021, following the World Health Organization’s international recommendations for COVID-19 surveillance (*20*). Thus, we only included patients that fit the case description for ILI or SARI in this study.

### Statistical Analysis

We used R version 4.0.3 (The R Foundation for Statistical Computing, https://www.r-project.org) and the supplementary R package, Moving Epidemic Method version 2.17 (https://github.com/lozalojo/mem), to process data and create epidemiologic curves. We generated average epidemic curves on the basis of percentages of SARI or ILI cases testing positive for influenza during each season. Then, we aligned the seasonal curves to generate an average curve, and set thresholds to define preepidemic, epidemic, and postepidemic periods. We defined the thresholds by calculating the upper limit of the 95% CI around the 30 highest weekly values. Our model also estimated sensitivity by correctly defining the epidemic period and specificity by correctly defining the nonepidemic period, and we calculated 95% CIs for the average season’s start date and duration (*21*,*22*).

## Results

During the prepandemic period, January 1, 2013–December 31, 2019, the 4S Network detected 74,726 ILI cases in community sites. Of those, 12,530 (17%) were randomly tested for influenza by rRT-PCR, and 3,157 (25%) were influenza-positive. During the same period, 776 SARI cases were hospitalized in sentinel sites and tested for influenza; 145 (19%) were positive (Table).

**Table:** RT-PCR test results demonstrating shifting patterns of influenza circulation during the COVID-19 pandemic, Senegal*

Testing per timeframe	No. (%) cases		
	ILI	SARI	Total
Prepandemic, 2013–2020			
No. cases enrolled	74,726	776	75,502
No. influenza RT-PCR performed	12,530 (1)	776 (100)	13,306 (17)
No. influenza-positive tests	3,157 (25)	145 (19)	3,302 (25)
Pandemic period, 2020–2022			
No. cases enrolled	19,030	1,352	20,382
No. influenza RT-PCR performed	2,593 (14)	1,352 (100)	3,945 (19)
No. influenza-positive tests	622 (24)	68 (5)	690 (17)
No. SARS-CoV-2 RT-PCR performed	1,409 (7)	1,129 (84)	2,538 (12)
No. SARS-CoV-2 positive tests	195 (14)	211 (19)	416 (16)

*Cases are from ILI and SARI surveillance during January 2013–July 2022. ILI, influenza-like illness; RT-PCR, reverse transcription PCR; SARI, severe acute respiratory illness

During the pandemic period, January 1, 2020–July 31, 2022, the 4S Network detected 19,030 ILI cases in community sites. Of those, 2,593 (14%) were randomly tested for influenza, of which 1,409 (54.3%) were also tested for SARS-CoV-2. Among tested samples, 622 (24%) were influenza-positive and 195 (14%) were SARS-CoV-2–positive. During the same period, 1,352 SARI cases were hospitalized in sentinel sites and tested for influenza, and 68 (5%) tested influenza-positive; 1,129 had combined SARS-CoV-2 and influenza testing, and 211 (19%) were SARS-CoV-2–positive (Table). Every specimen tested for SARS-CoV-2 was systematically tested for influenza, but the 2 pathogens were co-detected in only 1 patient.

### Local Influenza Epidemiology before COVID-19 Pandemic

**Figure 2:**
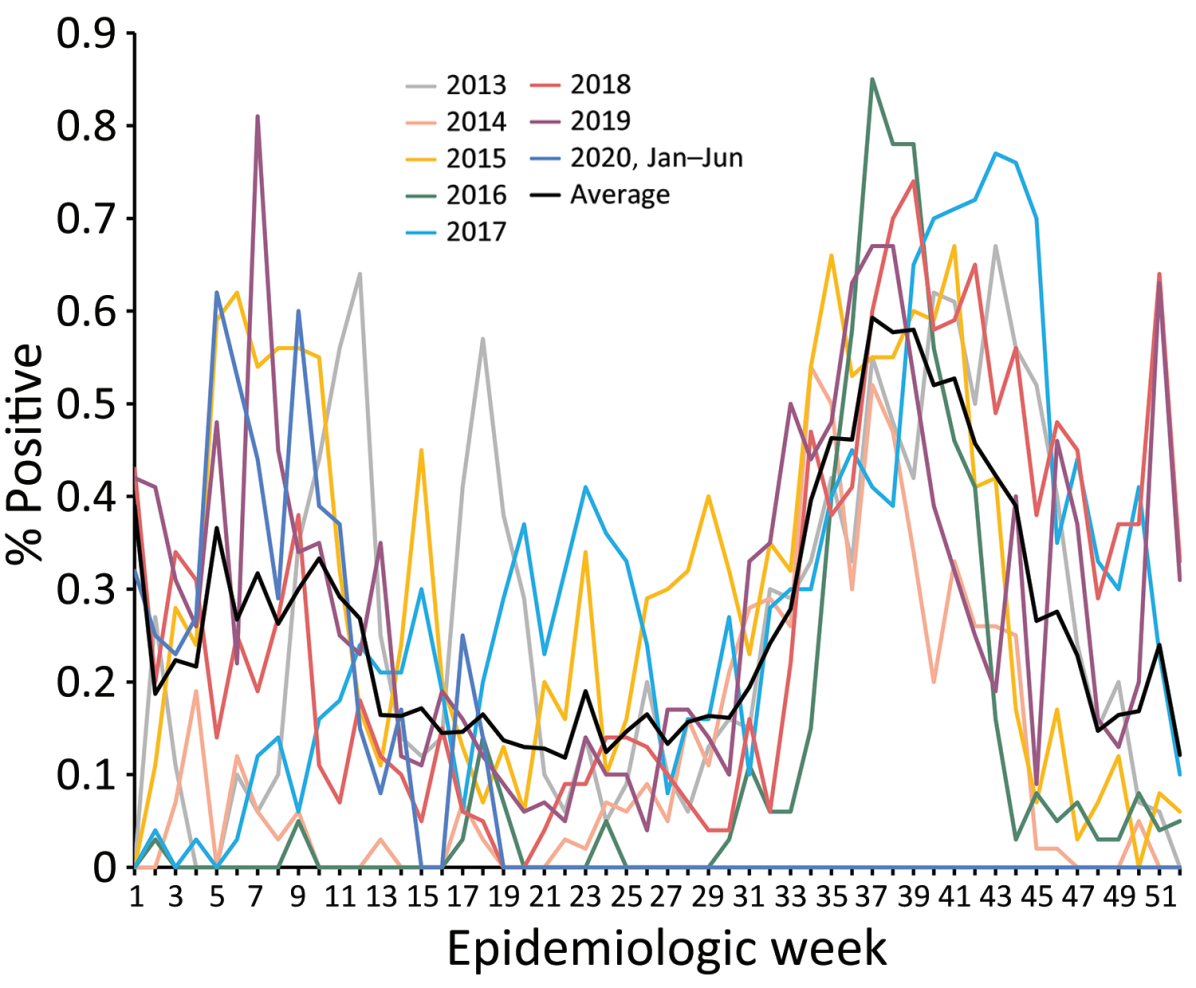
Prepandemic average epidemic curves used to demonstrate shifting patterns of influenza circulation during the COVID-19 pandemic, Senegal. Graphs show annual and overall average percentage of influenza-positive reverse transcription PCR tests per epidemiology week reported by the sentinelle syndromique du Sénégal (sentinel syndromic surveillance of Senegal), also known as the 4S Network, during January 2013–January 2020.

We found that, before the pandemic, Senegal had continuous circulation of influenza throughout the year and had 2 distinct seasonal peaks. The first peak typically occurred at the beginning of the year during epidemiologic week 5 (range week 1–13). The first peak typically ended around mid-April and had an average duration of 14 (95% CI 12–17) weeks and an average test-positive intensity peak of 34% (95% CI 10%–57%) of samples (Figure 2).

The second peak typically occurred during the second half of the rainy season, around August during epidemiologic week 31 (range week 27–36). That peak usually lasted until the end of November and had an average duration of 18 (95% CI 13–25) weeks and an average test-positive intensity peak of 61% (95% CI 47%–78%) of samples (Figure 2).

### Changes Observed in Seasonal Influenza during the COVID-19 Pandemic

**Figure 3:**
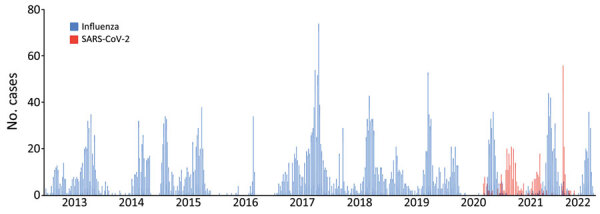
Average number of cases detected in a study of shifting patterns of influenza circulation during the COVID-19 pandemic, Senegal. Bars indicate number of reverse transcription PCR–positive tests for influenza and SARS-CoV-2 per epidemiology week reported by the sentinelle syndromique du Sénégal (sentinel syndromic surveillance of Senegal), also known as the 4S Network, during January 2013–July.

**Figure 4:**
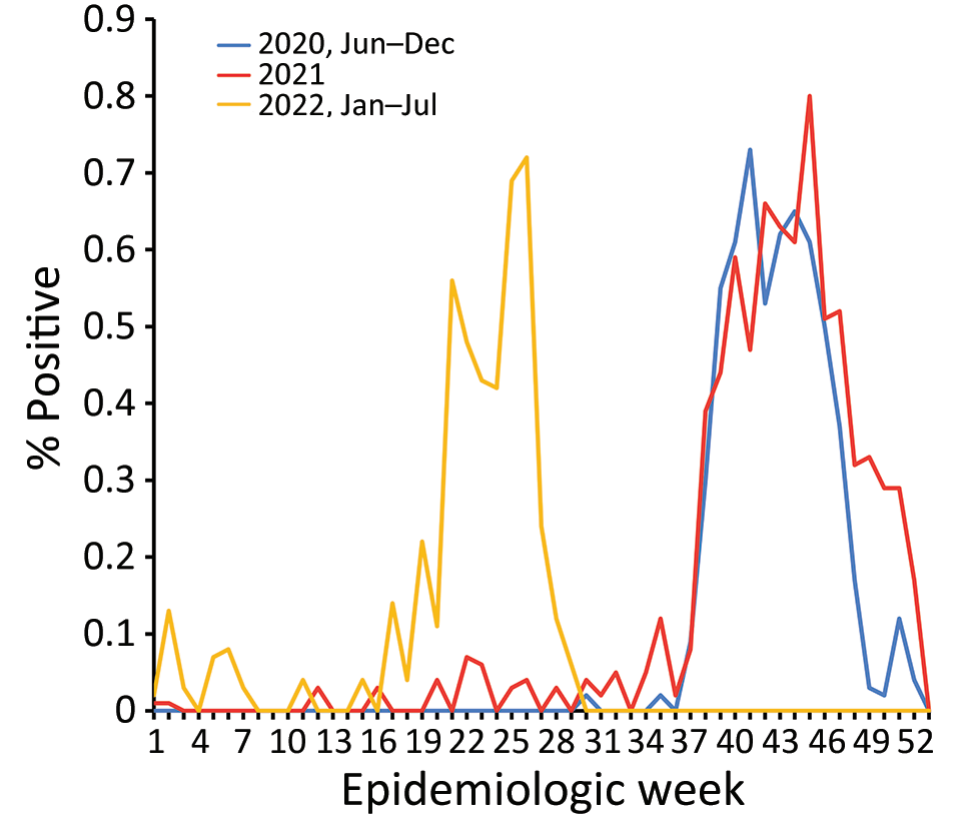
Average epidemic curves showing shifting patterns of influenza circulation during the COVID-19 pandemic, Senegal. Graphs show percentage of influenza-positive reverse transcription PCR tests per epidemiologic week reported by the sentinelle syndromique du Sénégal (sentinel syndromic surveillance of Senegal), also known as the 4S Network, during January 2020–December 2022.

**Figure 5:**
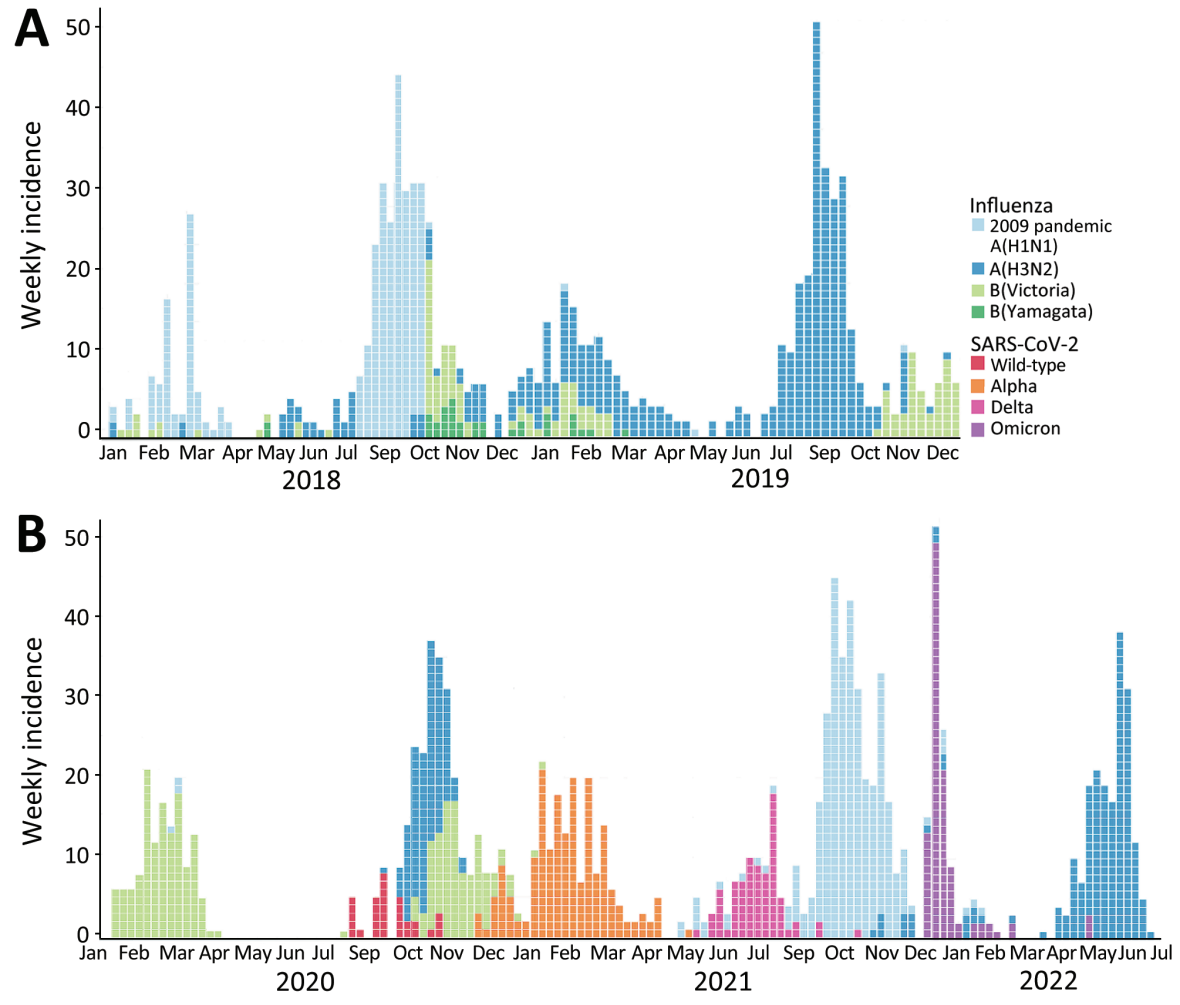
Number of reverse transcription PCR (RT-PCR)–positive samples per week in a study of shifting patterns of influenza circulation during the COVID-19 pandemic, Senegal. Data represent RT-PCR–positive tests per epidemiologic week reported by the sentinelle syndromique du Sénégal (sentinel syndromic surveillance of Senegal), also known as the 4S Network, including influenza subtypes and SARS-CoV-2 variants. A) Weekly influenza incidence during the prepandemic period, January 2018–2019. B) Weekly influenza and SARS-CoV-2 incidence during the pandemic period, January 2020–July 2022.

We observed that SARS-CoV-2 essentially transformed the biannual profile of influenza’s seasonal epidemic peaks in Senegal to a monophasic epidemic. During 2020, influenza circulation in Senegal seemed practically unperturbed. At the start of the year, influenza B (Victoria) virus peaked during January–March, after which a rainy season peak of influenza A(H3N2) and influenza B (Victoria) began during epidemiologic week 37, peaked at 73% of positive tests, and lasted for 11.5 weeks. SARS-CoV-2 started circulating in Senegal at the beginning of March 2020; the first case in Senegal was detected on March 2. However, systematic testing for SARS-CoV-2 was not added to the 4S Network until the beginning of June, which explains the low levels of SARS-CoV-2 detection during March–May 2020 (Figure 3). However, influenza surveillance continued during that period and revealed unusually low levels of influenza (Figures 4, 5).

During 2021, the expected beginning of the year influenza peak was completely absent. That period was marked by high levels of SARS-CoV-2 Alpha variant, after which an unmodified rainy season peak of 2009 pandemic influenza A(H1N1) started during epidemiologic week 37, peaked at 80% test-positivity, and lasted 10 weeks (Figures 4, 5).

The beginning of 2022 also was marked by the absence of the expected January–March influenza peak. That period also showed high levels of circulating SARS-CoV-2, but the Omicron variant dominated. Finally, an unexpected epidemic peak of influenza A(H3N2) was observed completely out of the usual period, starting in May during epidemiologic week 17 when influenza activity is usually the lowest in Senegal, and ending in July, during epidemiologic week 29, with a maximum peak of 71% test positivity (Figures 4, 5). Of note, influenza B (Yamagata) has practically disappeared in Senegal since June 2020; the last 2 cases were detected in January 2021.

## Discussion

Before the COVID-19 pandemic, the dynamics of influenza in Senegal mostly followed the various patterns seen in tropical regions, showing year-round low-level circulation and increased activity during the rainy seasons (*1*,*2*). Senegal also had a typical smaller influenza peak at the start of the year (Figure 5, panel A).

Influenza’s seasonal patterns and variability across different climate zones is still only partially understood (*23*). Among other factors, dry and cold weather conditions appear to promote influenza circulation in temperate regions (*23*–*25*), which is supported by in vitro and in vivo models (*24*). However, weather conditions do not account for observations made in tropical areas where circulation often peaks around months with the highest temperature and humidity levels (*25*–*27*).

Many other seasonally dependent factors influence influenza’s circulation: fluctuations in host competence and immune response; changes in population behavior, such as school attendance; and the amount of time spent indoors (*23*). In Senegal, the rainy season is a period when most of the population is frequently forced to stay at home because of violent rainfall that disrupts normal traffic and human mobility patterns. The increase in indoor human contact and the return to school of a predominantly young population during the same season certainly contribute to the observed rainy season peak in Senegal and possibly in other countries (*26*).

Increased indoor contact does not account for the peak seen at the start of the year, which is the middle of Senegal’s dry season. However, school schedules and international travel might be implicated in the peak. Children returning to school increase influenza circulation. In addition, many persons travel to Europe, which usually experiences its annual influenza season at that time. Travel between Senegal and northern Europe peaks during the end of the year, when persons from Senegal return from visiting their families in Europe during the winter holidays and tourists from Europe who favor the dry season travel to Senegal to visit. The role of international travel on the January–March influenza peak is also suggested by the absence of influenza at the beginning of 2016, which corresponded to the period of the Ebola epidemic in West Africa that resulted in travel restrictions (Figure 3). Among the NPIs used during the COVID-19 pandemic, travel restrictions might have had a role in reshaping the biannual seasonality of influenza in Senegal into a more monophasic epidemic.

However, Senegal did not have a biannual influenza epidemic profile until after the implantation of the pandemic H1N1 2009 strain in the territory in 2010 (*27*). That observation suggests that climate, host immunity, and behavior might not be the only contributing factors to the seasonality of influenza circulation and that emergence of new competitive viral strains can also have a prolonged effect on periodic influenza circulation patterns.

### Changes Observed during COVID-19 Pandemic

During 2020–2021, countries in the Southern Hemisphere that have temperate climates, such as Australia and South Africa, reported close to zero influenza circulation, and influenza remained mostly absent until 2021 (*28*). In the Northern Hemisphere, the influenza seasonal peak of the 2020–21 winter was also absent (*29*,*30*). Those periods showed high levels of SARS-CoV-2 circulation during the second pandemic wave of the Alpha variant and subsequent reinforcement of NPIs (*31*).

In Senegal, at the end of March 2020, face masks became mandatory in public places, public gatherings were forbidden, international flights were closed, and a curfew was put in place (*32*). Those measures were gradually alleviated at the end of July 2020, when curfew hours were lightened and international flights were reopened, but Senegal maintained a high level of border control. A noticeable reduction of population mobility was recorded during March 2020–March 2021 (*33*).

The arrival of SARS-CoV-2 in Senegal had noticeable effects on local influenza circulation. Unlike reports from temperate regions, only the expected January–March influenza peak was affected in Senegal, but the main rainy season peaks stayed unperturbed in their timing and intensity (Figure 5, panel B). That finding could be partially explained by concurrent reinforcement or alleviation of NPIs. However, influenza activity in Senegal did not seem well correlated with local NPI reinforcement. Senegal noticeably alleviated its contact restriction measures around March 2021 (*34*), as illustrated by the noticeable drop in its estimated COVID-19 Stringency Index (*35*) and the concomitant rise in the population’s mobility, as estimated by Google’s COVID-19 Community Mobility Reports (*33*). That timeline does not account for influenza’s recorded activity during the study period.

The abnormally low levels of influenza in the early months of 2021 and 2022 might be explained by the link between the expected start of the year peak and the winter peak usually seen in the Northern Hemisphere. That start of the year peak would be more dependent on international travel, as described, which might explain the unbalanced effect of the COVID-19 pandemic on influenza circulation in Senegal.

Deciphering the underlying causes of those shifts is challenging because the pandemic affected every level of the human ecosystem. The role of social distancing and other NPIs is undeniable because it necessarily affects the number of potentially contaminating social encounters. However, as those measures were gradually alleviated, influenza and SARS-CoV-2 continued to circulate alternately. The observed reciprocal nature of influenza and SARS-CoV-2 circulation, which is easier to visualize in Senegal’s tropical setting, calls into question the prevailing role of NPIs and travel restrictions and invites us to search for other contributors.

Negative viral interference or viral competition—that is, the transient inhibitory effects that a virus can have on secondary infection by other viruses at the host level, essentially through sustained interferon pathway activation—s an old concept that has been studied and confirmed by in vitro and animal models (*36*,*37*) and has been supported by epidemiologic observations and statistical modeling (*36*,*38*,*39*). Although the concept is still controversial, some argue that rhinoviruses might have participated in the dissipation of first the wave of the 2009 pandemic influenza A(H1N1), for instance (*40*). Viral interference between SARS-CoV-2 and influenza has also been studied experimentally (*41*,*42*) and is supported by epidemiologic data (*43*,*44*). The implication of negative viral interference on influenza circulation is further supported by the very low levels of co-detection noticed at the patient level, only 1 case of co-detection out of 2,538 tests performed during our study period. Cases of SARS-CoV-2 and influenza co-infections have been reported in the literature but seem to be rare (<1%) (*43*).

The surveillance network used in this study has certain advantages, such as wide geographic coverage and use of community and hospital settings. However, the 4S Network exclusively provides information on symptomatic patients because of its focus on syndromic surveillance; thus, the network omits some local influenza and SARS-CoV-2 epidemiologic features. Also, locations of sentinel sites might have underrepresented populations from remote areas, especially in the northeastern and southeastern parts of Senegal, the most underpopulated areas of the country.

Because the network provides close to real time information, we were able to integrate recent data and cover more post–COVID-19 influenza seasons. Thus, we could offer a broader view of the effects of SARS-CoV-2 on influenza circulation in Senegal, which has public health implications that seem to be ongoing (*5*).

During March–June 2020, which corresponds to the first SARS-CoV-2 pandemic wave in Senegal, the activity of the surveillance system was drastically decreased. At that time, COVID-19 tests were not available, and local healthcare providers from sentinel sites were asked by the ministry of health to train colleagues in neighboring districts to perform nasopharyngeal sampling and conduct local case investigations. Nevertheless, routine influenza surveillance was not completely abandoned during that period, and approximately one third of the usual number of samples were sent for influenza testing. Therefore, the absence of influenza notifications during the first SARS-CoV-2 wave was not only because of a lack of testing but also because of low levels of concurrent influenza circulation, consistent with what was seen later.

Data regarding influenza and SARS-CoV-2 circulation in tropical regions are scarce. In addition, our data are limited to a small geographic area and timeframe, just 2 years of co-circulation. Distinguishing crucial and durable changes in influenza’s circulation patterns requires a broader scope. Therefore, data from other tropical countries and over longer periods of time are needed to clarify the effects of the COVID-19 pandemic on influenza circulation patterns in tropical regions.

Many questions on how influenza’s seasonality will be affected in the long term remain. Influenza seasonality is probably intimately linked to SARS-CoV-2 and its potential for becoming a seasonal virus. In addition, SARS-CoV-2 could interfere with influenza circulation through broad population behavioral responses and host level immunologic and virologic determinants.

In conclusion, although NPIs and travel restrictions most certainly were predominant factors in the disruption of influenza circulation in 2020 and early 2021, those now seem insufficient to account for the more recent observations made in Senegal and other countries. Thus, the role of viral interference in reshaping influenza seasonality should be considered and included in future virologic and epidemiologic studies.
